# High-Performance Planar-Type Photodetector on (100) Facet of MAPbI_**3**_ Single Crystal

**DOI:** 10.1038/srep16563

**Published:** 2015-11-13

**Authors:** Zhipeng Lian, Qingfeng Yan, Qianrui Lv, Ying Wang, Lili Liu, Lijing Zhang, Shilie Pan, Qiang Li, Liduo Wang, Jia-Lin Sun

**Affiliations:** 1 Department of Chemistry, Tsinghua University, Beijing 100084, China; 2 Key Laboratory of Functional Materials and Devices for Special Environments, Xinjiang Technical Institute of Physics & Chemistry, Chinese Academy of Sciences, 40-1 South Beijing Road, Urumqi 830011, China; 3 Collaborative Innovation Center of Quantum Matter, State Key Laboratory of Low-Dimensional Quantum Physics, Department of Physics, Tsinghua University, Beijing 100084, China

## Abstract

Recently, the discovery of organometallic halide perovskites provides promising routes for fabricating optoelectronic devices with low cost and high performance. Previous experimental studies of MAPbI_3_ optoelectronic devices, such as photodetectors and solar cells, are normally based on polycrystalline films. In this work, a high-performance planar-type photodetector fabricated on the (100) facet of a MAPbI_3_ single crystal is proposed. We demonstrate that MAPbI_3_ photodetector based on single crystal can perform much better than that on polycrystalline-film counterpart. The low trap density of MAPbI_3_ single crystal accounts for the higher carrier mobility and longer carrier diffusion length, resulted in a significant performance increasement of MAPbI_3_ photodetector. Compared with similar planar-type photodetectors based on MAPbI_3_ polycrystalline film, our MAPbI_3_ single crystal photodetector showed excellent performance with good stability and durability, broader response spectrum to near-infrared region, about 10^2^ times higher responsivity and EQE, and approximately 10^3^ times faster response speed. These results may pave the way for exploiting high-performance perovskites photodetectors based on single crystal.

Photodetectors which can convert light signals into electrical ones instantaneously have sparked wide interests because of their important applications, such as video imaging, optical communication, environmental monitoring, and biomedical sensing^1^,^2^,^3^,^4^. The recent discovery of solution-processed organometallic halide perovskites (MAPbX_3_, X = I, Br, Cl) provides promising routes for fabricating optoelectronic devices with low cost and high performance^5^,^6^,^7^,^8^. Among these organometallic halide perovskites, methylammonium lead triiodide (MAPbI_3_) has been the spotlight owing to its appropriate direct band gap^9^, large absorption coefficient^6^, long-range balanced electron- and hole-transport lengths^10^,^11^, and high charge carrier mobilities^11^,^12^,^13^. Park and Graetzel reported the strong photocurrent multiplications of the MaPbI_3_ which enables its application as a photodetector^14^. Until now, several significant efforts to fabricate novel high-performance MAPbI_3_ photodetectors, such as perovskite-graphene hybrid photodetector^15^, perovskite nanowires-based photodetectors^16^,^17^, and highly stable perovskite photodetector^18^, etc. have been made. The performances of several recently reported pure MAPbI_3_ based photodetectors are listed in Supplementary Table S1. It can be seen that photodetectors based on organometallic halide perovskite MAPbI_3_ demonstrate the advantages of high-responsivity, high quantum efficiency, and fast response speed at low driving voltage.

In general, these MAPbI_3_-based photodetectors can be classified into two structural types: planar-type and vertical-type. Xie *et al.*^19^ reported the first MAPbI_3_ photodetector, which is a typical planar-type one. The MAPbI_3_ polycrystalline film photodetector (MPFP) was found sensitive to a broadband response spectrum ranging from the ultraviolet light to entire visible light, showing a high photo-responsivity (R) of 3.49 A W^−1^, 0.0367 A W^−1^, and an external quantum efficiency (EQE) of 1.19 × 10^3^%, 5.84% at 365 nm and 780 nm under a bias voltage of 3 V, respectively. Dong *et al.*^20^ demonstrated a vertical-type highly efficient photodetector based likewise on MAPbI_3_ polycrystalline film, showing a broadband response spectrum that ranges from the 300 nm (UV) to 800 nm (NIR). The gain of their MAPbI_3_ photodetectors was influenced by the MAPbI_3_ layer thickness and achived the maximum when the thickness was between 360 and 410 nm. A very high responsivity of 242 A W^−1^ at low bias (−1 V) was attained by this vertical-type photodetector. Compared with the planar-type photodetectors, the elaborate vertical-type devices require more patterning steps for fabrication. On account of the inevitable non-radiative defects iccured by these steps at the edges of the layers, the device performance will be detrimentally affected^21^. However, as a matter of fact, the vertical-type MPFPs could perform much better than the planar-type ones, demonstrating higher gain and quicker response. The reason, on the one hand, lies in the fact that the electrode spacing plays an important role in making these differences. As for planar-type MPFPs, the spacing is typically tens of micrometers long, while that of the vertical-type MPFPs is usually tens to hundreds of nanometers long. The lenghth of the former is influenced by the bridging-gap on the substrate^19^, and that of the latter is determined by the film thickness. As a result, the electrons and holes in the planar-type devices have to move much longer than those in the vertical-type ones to the electrodes^22^. On the other hand, numerous defects and extensive disorders in MAPbI_3_ polycrystalline film antagonize the charge migration process. All the problems mentioned above hinder the MAPbI_3_ photodetectors from ultilizing intrinsic properties to achieve the optimal performance, especially for planar-type devices.

To improve the performance of MAPbI_3_ photodetectors, it is necessary to reduce the defect density by increasing the crystalline quality of MAPbI_3_ film, or perhaps more directly, to fabricate photodetectors based on single crystals MAPbI_3_. Recently, single crystals of perovskites MAPbI_3_ have shown remarkably low trap density and excellent charge transport properties. Shi *et al.*^23^ observed low trap-state density on the order of 10^9^ to 10^10^ per cubic centimeter and long charge carrier diffusion lengths exceeding 10 micrometers in MAPbX_3_ single crystals. Dong *et al.*^24^ reported that the diffusion lengths in MAPbI_3_ single crystals could exceed 175 μm under 1 sun illumination and exceed 3 mm under weak light for both electrons and holes, which far exceeds the value measured from polycrystalline MAPbI_3_ films (typically smaller than 1 μm)^10^,^11^. These results potentially suggest that the optoelectronic device based on single-crystalline perovskite could perform much better than that on the polycrystalline counterpart. Thus, remarkable performance improvement is expected in the planar-type perovskite photodetector regardless of the ten-micron-long electrode spacing. Additionally, degradation of MAPbI_3_ has always been a notorious drawback for their real application in optoelectronic devices. The MAPbI_3_ polycrystalline film device without modification usually wears off very quickly when exposed to ambient air^25^,^26^. Many efforts have been made to enhance the durability of perovskite polycrystalline film devices, such as the smart and simple way of spin-coating with a CYTOP layer on perovskite film^18^. Nevertheless, the MAPbI_3_ single crystal may provide better stability, since it averts the prejudicial effects of grain boundaries and a large number of surface defects in polycrystalline film.

In this work we propose a planar-type photodetector fabricated on the (100) facet of an MAPbI_3_ single crystal. We demonstrate that MAPbI_3_ single crystal photodetector (MSCP) can perform much better than the devices based on polycrystalline film. Although it was fabricated as the simplest device without elaborate eletrode decoration, the MSCP demonstrated high responsivity of 953 A W^−1^ and EQE of 2.22 × 10^5^% at 1 V applied voltage when illuminated with the weakest detectable 532 nm light of 2.12 nW cm^−2^. When exposed to a relative strong light at 1 mW cm^−2^, compared with the similar planar-type photodetector based on polycrystalline (R: 0.0197 A W^−1^, EQE: 4.59%), the MSCP showed about 100 times higher responsivity of 2.55 A W^−1^ and EQE of 5.95 × 10^2^%. Additionally, the MSCP showed excellent performance with good stability and durability, broader response spectrum to near-infrared region and approximately three orders of magnitude faster response speed.

## Results

**Figure 1:**
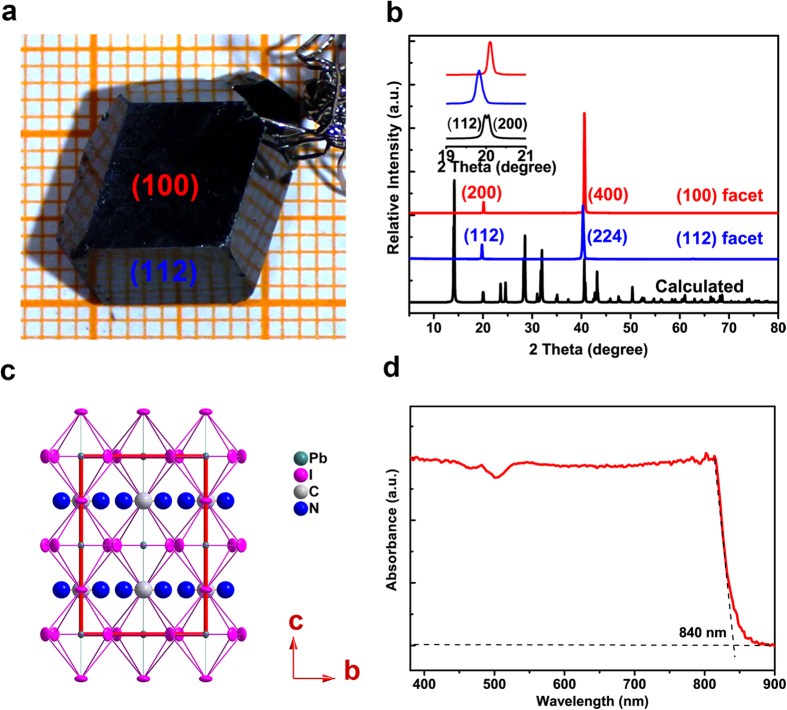
Characterization of as-grown MAPbI_3_ bulk single crystal. (**a**) Photograph of a MAPbI_3_ bulk single crystal grown through the BSSG method. Natural facets of the MAPbI_3_ bulk single crystal were indexed. The unit length of each grid on the coordinate paper is 1 mm. (**b**) XRD patterns of the rhombic (red) and the parallelogram (blue) natural crystallographic facets of a MAPbI_3_ single crystal. The calculated powder XRD pattern (black) is also shown as a reference. The inset shows the details of distinguishing (100) and (112) facets. (**c**) Drawing of the structure of (100) facet-oriented MAPbI_3_ crystal (partial N atoms are made invisible in order to expose the C atoms). (**d**) UV-visible absorption spectrum for a (100)-cut 1 mm-thick MAPbI_3_ single crystal plate.

Centimeter-sized MAPbI_3_ bulk single crystals were grown via a bottom-seeded solution growth (BSSG) method. Seed crystals were first prepared according to Poglitsch and Weber’s method^27^. It was found that MAPbI_3_ was quite easy to crystallize during the seeded growth process. Some deposits appeared in the bottom of the flask and tended to adhere to the surface of the growing seed during the cooling process. The formation of many nucleuses obviously antagonizes the growth of large bulk single crystal of MAPbI_3_. To eliminate such a negative effect, a platinum wire was utilized to fix and prop the seed crystal up to separate the seed crystal from contacting the bottom of the flask. A clean bulk single crystal was obtained after lowering the temperature of the growth solution from 373 K to 330 K over the course of 15 days. A MAPbI_3_ single crystal with the dimension of 12 mm × 12 mm × 7 mm is shown in Fig. 1a with the oriented natural facets labelled. X-ray single crystal structural analyses were performed at room temperature and the space group was confirmed as I4/mcm (140), which was consistent with the report in 1987^27^. For MAPbI_3_ single crystal, three-dimensional Pb-I links form the integral structural framework, and the MA^+^ ion is in a cage made by four PbI_6_ octahedra. Details of structural description is presented in the supplementary material (Supplementary Table S2 and Figure S1–2). A comparison of XRD patterns of the rhombic and the parallelogram natural crystallographic facets of a MAPbI_3_ single crystal is presented in Fig. 1b, where the calculated polycrystalline XRD pattern was provided as a reference. The inset in Fig. 1b shows the detail when 2 theta is around 20 degree. The obvious difference between the two patterns is a direct guidance to identify the rhombic and the parallelogram natural crystallographic facets as (100) and (112) facets, respectively. Sharp and symmetric peak pattern of each facet indicates the good quality of the MAPbI_3_ single crystal. As presented in Fig. 1c, the structural drawing of (100) facet of MAPbI_3_ crystal suggests the modeled transporting tunnel for photo-generated electrons and holes. Optical properties were measured on a (100)-cut MAPbI_3_ single crystal plate with 1 mm in thickness (Supplementary Figure S3). It was found that the MAPbI_3_ single crystal presents a broad band absorption extending to 840 nm (Fig. 1d), implying potentiality of applying this material in near-infrared optoelectronic devices. For the semiconductor material, the optical absorption is closely related to the energy gap between the conduction and the valence band. Through first principle computation and experimental study, MAPbI_3_ exhibits the optical features of a semiconductor with direct band gap^28^,^29^. Supplementary Figure S4 shows the transmission spectrum of as-prepared MAPbI_3_ single crystal plate at room temperature. The transmission intensity maintains high from 887 nm to 1200 nm and gradually decreases below 887 nm with a cut-off wavelength of 813 nm. As presented in the inset of Supplementary Figure S4, the optical band gap of MAPbI_3_ single crystal was deduced through extrapolation method and calculated to be 1.48 eV, which is consistent with Tao and co-workers’ report^30^, but lower than 1.5 ~ 1.6 eV as reported previously in thin polycrystalline film^31^,^32^. Remarkably, optical absorption characterization based on a single crystal clarifies the lower but more accurate band gap evaluation for MAPbI_3_ material, eliminating the impacts of other factors such as grain boundaries, substrates and scaffolds. To conclude, MAPbI_3_ single crystal possesses a broader absorption band. Therefore, it is more advantageous to act as light-harvester or photodetector than polycrystalline film. In addition, Raman vibrational spectrum of MAPbI_3_ single crystal in the low-frequency region at room temperature was collected and assigned (Supplementary Figure S5).

**Figure 2:**
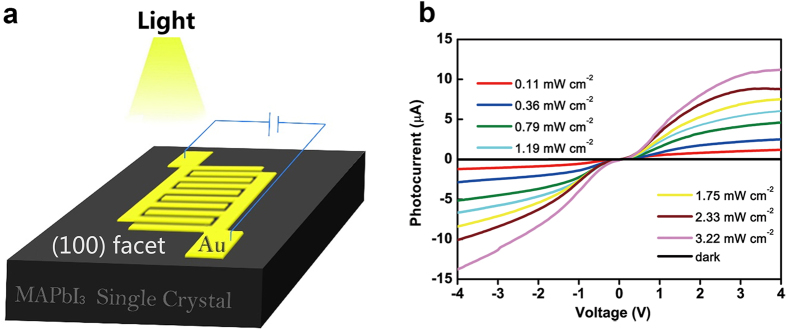
Structure and I~V properties of as-fabricated MSCP irradiated by a 532 nm laser. (**a**) Schematic illustration of the planar-type photodetector fabricated on the (100) facet of a MAPbI_3_ single crystal. (**b**) Photocurrent versus voltage curves under various irradiation densities.

As schematically illustrated in Fig. 2a, a pair of interdigitated Au-film electrodes were deposited on the (100) facet of MAPbI_3_ single crystal to form a planar-type photodetector. The bridging-gap of electrodes width was about 20 μm, while the effective illuminated area of MAPbI_3_ single crystal was about 1.19 × 10^−7^ m^2^ (Supplementary Figure S6). To study the photo-electric convertibility of our device, I ∼ V curves under different illumination power were measured by using a 532 nm laser as the light source with the power densities varying from 0.11 to 3.22 mW cm^−2^ (Fig. 2b). All measurements were performed in air at room temperature. The dark current was plotted as a reference, which was as low as 11 nA at 4 V bias. When exposed to light, the photocurrent increased dramatically. The I-V curves show a nonlinear and asymmetrical behavior, indicating that a Schottky contact between MAPbI_3_ single crystal and Au electrodes was formed. It is observed that the rising rate of photocurrent increases first and then decreases, implying that it may undergo a saturation effect of photocurrent with bias voltage increasing. This phenomenon may arise from the built-in electric fields effect. The charging and discharging processes by switching power supply of 1 V and 0 V under 0.79 mW cm^−2^ illumination were recorded in Supplementary Figure S7, and the existence of the built-in electric fields was expounded in the supplementary material. A high photocurrent of 11.2 μA was achieved at −3 V bias under 3.22 mW cm^−2^ illumination.

Two crucial parameters for photodetectors, i.e. spectral responsivity (R) and EQE, were measured and calculated. The spectral responsivity, defined as the ratio of photocurrent to incident light intensity, can be expressed by:


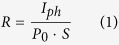


where *I*_ph_ is the difference between the illuminated current and dark current (*I*_ph_ = *I*_illuminated_ − *I*_dark_), *P*_0_ is the irradiance power density, and *S* is the effective illuminated area^33^,^34^. It is also very important for a photodetector to have a high conversion rate from photons to electrons/holes, or high EQE, which can be expressed by the equation:


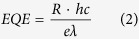


where *R* is the spectral responsivity, *h* represents the Planck’s constant, *c* stands for the velocity of light, *e* is the electronic charge and *λ* is the wavelength of incident light^19^,^35^.

**Figure 3:**
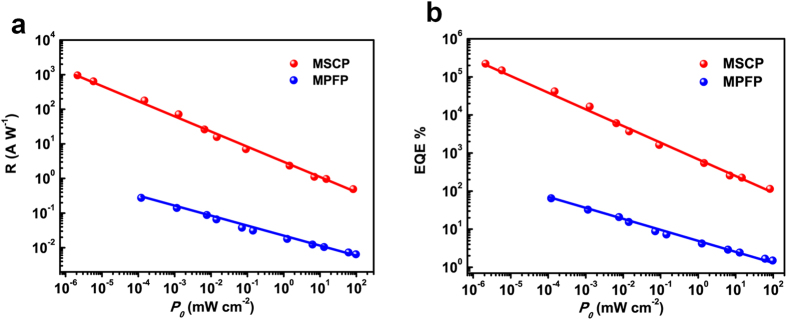
A comparison of irradiance power-dependent photoresponse characteristics, responsivity R (**a**) and external quantum efficiency EQE (**b**), for as-fabricated MSCP and MPFP. The light source was a 532 nm laser and all measurements were performed at a fixed bias of 1 V. The irradiance power densities for each device are in a range from the lowest detectable value to about 10^2^ mW cm^−2^.

To illustrate the superiority of single crystal MAPbI_3_, MPFP devices were fabricated as reference for comparisons. Considering that the value of responsivity and EQE strongly depends on the applied voltage, light power and device structure, identical interdigitated Au-film electrodes was deposited on the surface of MAPbI_3_ thin-film, structurally the same as MSCPs on (100) crystal facet, as shown in Supplementary Figure S8. Furthermore, the irradiance power-dependent photoresponse characteristics, such as R and EQE values for both MSCP and MPFP were evaluated at a fixed 1 V bias, as shown in Fig. 3a,b. One can see that both the R and EQE decreased as the irradiance increased, thus the highest values for R and EQE could be measured at the lowest detectable irradiance power. It is worth pointing out that for each device of MSCP and MPFP, the irradiance power densities were intentionally selected ranging from the lowest detectable value to about 10^2^ mW cm^−2^. The MSCP was found to achieve better detection thresholds than MPFP. The lowest detectable irradiance power density was evaluated to be 2.12 nW cm^−2^ for the former, under which the highest R value of 953 A W^−1^ and and EQE of 2.22 × 10^5^% were ahieved. Below 120 nW cm^−2^, the photocurrent signal of MPFP could no longer be observed as it was covered by the noise and dark current. Therefore the highest R and EQE values for MPFP at 1 V voltage were calculated to be 0.28 A W^−1^ and 64.64%, respectively. Moreover, under illumination with the same irradiance power density of 1 mW cm^−2^, the R values for MSCP and MPFP were estimated to be 2.55 A W^−1^ and 0.0197 A W^−1^, while the corresponding EQE values were calculated to be 5.95 × 10^2^% and 4.59%, respectively. Clearly the results demonstrated that the responsivity and EQE for MSCP are more than two orders of magnitude higher than those of MPFP.

At the same voltage, the photocurrent increased gradually as their radiance intensity increased. The relationship between the photocurrent and the irradiance power density can be fitted by the power law:


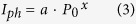


where *I*_ph_ represents photocurrent, 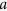 is constant, *P*_0_ represents irradiance power density, and *x* is the exponential term. At a bias of 1 V, the irradiance-dependent photocurrent was plotted and fitted by *I*_ph_ ∼ *P*_0_^0.632^ (Supplementary Figure S9). Moreover, linear dynamic range (LDR, typically quoted in dB), another figure-of-merit for photodetectors, was evaluated for MSCP. As depicted in Supplementary Figure S10, under illumination at 532 nm, the LDR for our MSCP was calculated to be 76 dB.

**Figure 4:**
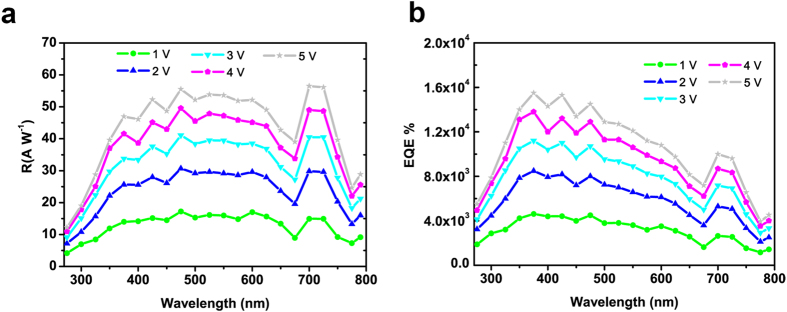
Wavelength-dependent properties of as-fabricated MSCP irradiated by monochromatic light from Xe lamp. Spectral responsivity R (**a**) and external quantum efficiency EQE (**b**) of MAPbI_3_ single crystal photodetector at different wavelengths from 275 nm to 790 nm at various bias from 1 V to 5 V.

Next, the spectral photoresponse was measured with a 300 W Xe lamp equipped with a manual monochromator. The MSCP shows a broadband detection characteristic from 275 nm to 790 nm, with a high R of around 10 A W^−1^ and EQE of over 10^3^% even at a relatively low bias voltage of 1 V (Fig. 4). The corresponding irradiance power density (*P*_*0*_) at each measured wavelength were depicted in Supplementary Figure S11. It is notable that the R and EQE of our MSCPs sustained high till the near-infrared wavelength of 790 nm, while that of MPFPs decreased sharply from 725 to 800 nm. These results indicate that MSCP possesses a broader spectral detectivity than MPFP. It should be noted that the characterization of R and EQE over 790 nm are not successful due to the limitation of the monochromator used. Nevertheless, a broadband detection cut-off towards 840 nm is expected, considering the absorption characteristics of the MAPbI_3_ single crystal (Fig. 1d). The MSCP presented a maximum EQE value of 1.55 × 10^4^% at 5 V with 375 nm 3 × 10^−3^ mW cm^−2^ irradiance, and the corresponding responsivity was calculated to be 47.1 A W^−1^. Additionally, both the R and the EQE decreased when the voltage varied from 5 V to 1 V.

**Figure 5:**
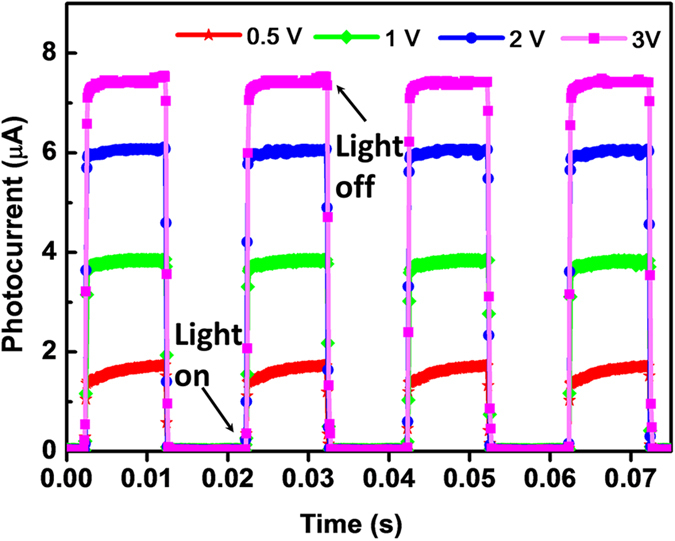
Time-dependent photocurrent response of MSCP. Photocurrent decay and rise at different voltages of 0.5, 1, 2, and 3 V with on-and-off light (532 nm, 1.75 mW cm^−2^).

**Figure 6:**
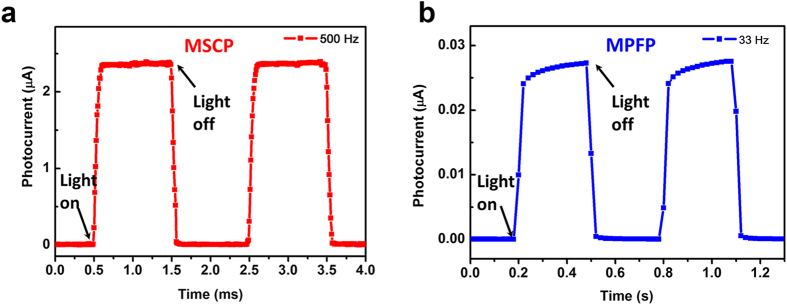
A comparison of transient photocurrent characteristics for as-fabricated MSCP and MPFP. (**a**) Transient photocurrent response for the MSCP with on-and-off light with a switching frequency of 500 Hz. (**b**) Transient photocurrent response for the MPFP with a switching frequency of 33 Hz. The measurements were conducted under 0.79 mW cm^−2^ illumination using a 532 nm laser.

**Figure 7:**
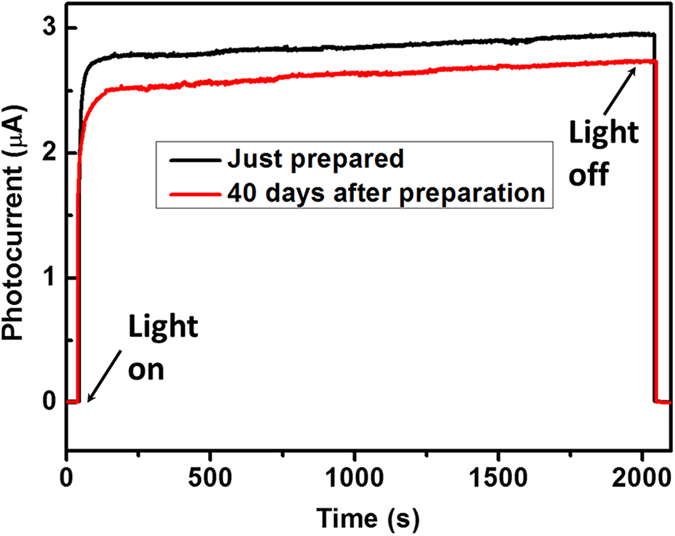
Photocurrent stability and durability of the as-fabricated MSCP. The black line was measured as soon as the device was fabricated, and the red line was measured after storing 40 days in a desiccator at room temperature. The test conditions were both under 532 nm 0.79 mW cm^−2^ laser illumination at 3 V bias.

Figure 5 shows the photo-switching characteristics of the MSCP at different applied voltages of 0.5, 1, 2, and 3 V, respectively, and excellent repetition of photocurrent was observed under photo-switching conditions. A peak photocurrent of 7.6 μA was achieved at 3 V bias, exhibiting a good photocurrent on/off ratio of 224. A fast response speed to light switch is much desired for the purpose of photodetector application. To further demonstrate the response speed of the MSCP, the high speed features of the device were measured by an optical chopper of 500 Hz under 0.79 mW cm^−2^ illumination using a 532 nm laser (Fig. 6a). Defined as the time taken from the initial photocurrent to 90% increase and decrease, the rise and decay times for our MSCP were calculated. The rise time was 74 μs after triggering and the decay time was 58 μs after irradiation terminated. Similarly, as a comparison, by utilizing a optical chopper of 33 Hz the rise and decay times for MPFP were measured to be 52 and 36 ms (Fig. 6b), which were comparable to the literature^19^. The above results reveal that the response speed for MSCP was three orders of magnitude faster than MPFP. Figure 7 presents the stability and durability of the MSCP. Within the scanning time of about 2,000 s, no obvious degradation of photocurrent was observed for each curve measured before and after 40 days’ storage in a desiccator at room temperature, indicating excellent photocurrent stability of the MSCP device. Besides, it still kept a high photocurrent of 2.65 μA that only fell by 6% after 40 days’ storage, meaningfully revealing a good durability of the natural MSCP device without any modification. However, it’s also worth mentioning that kept in test situations (atmosphere with a relative humidity of about 45 per cent), the MPFP device wore off within a few days while the MSCP still worked for a much longer time.

## Discussion

Previous experimental studies of MAPbI_3_ optoelectronic devices, such as photodetectors and solar cells, are normally based on polycrystalline films. In the current study, we first revealed the much higher performance, based on single crystal, of the MAPbI_3_ photodetector. It is crucial to understand the nature of the high-performance of the as-fabricated MSCP. It undergoes various photophysical processes and dynamic interplays in bare MAPbI_3_ as the photoexcitation is triggered, such as absorption, generation of electron-hole pairs, charge transfer and recombination^36^. Firstly, the specific crystal structure and electronic structure of MAPbI_3_ provide appropriate chance for harvesting light while ensuring high efficiency. Bulk single crystal may play a better role in absorbing photons because it is much thicker than a thin-film. Once exposed to the illumination with a larger photoenergy than the band gap, MAPbI_3_ absorbs photons to generate a large number of electron-hole pairs [hν → e^−^ + h^+^]^19^. An applied voltage bias provides the local electric field for both MAPbI_3_ single crystal and its polycrystalline film counterpart, leading to separate the photogenerated electrons and holes and decrease the electron-hole recombination rates, which is of great importance for the high gain of the photodetector. After excitons dissociation, the electrons would migrate to the conduction band of MAPbI_3_ and further to the cathode, while the holes would flow to the valence band and further to the anode. During the carrier migration process, the resistance of the transporting tunnels made a difference between in MAPbI_3_ polycrystalline film and in MAPbI_3_ single crystal. For the former, numerous defects and extensive disorders impede the carrier migration and hinder the MAPbI_3_ photodetectors from achieving the optimal performance. As to the latter, the low trap density, which was dramatically reduced by 2 to 3 orders of magnitude^23^, reasonably give rise to the higher carrier mobility and much longer carrier diffusion length. As a result, significant performance improvements were eventually acheived for the MSCP.

In summary, centimeter-sized MAPbI_3_ bulk single crystals were successfully grown via the BSSG method. Optical characterization demonstrated excellent absorption in the visible region with an absorption cut-off edge towards 840 nm, which was broader than that of MAPbI_3_ polycrystalline film. A planar-type photodetector was fabricated on the (100) facet of MAPbI_3_ single crystal for the first time. The lowest detectable irradiance power density for MSCP was evaluated to be 2.12 nW cm^−2^, under which the highest R value of 953 A W^−1^ and and EQE of 2.22 × 10^5^% were ahieved. When exposed under 1 mW cm^−2^ illumination of 532 nm, the R values for MSCP and MPFP were estimated to be 2.55 A W^−1^ and 0.0197 A W^−1^, while the corresponding EQE values were calculated to be 5.95 × 10^2^% and 4.59%, respectively, demonstrating more than two orders of magnitude higher responsivity and EQE for MSCP. The transient photocurrent measurement showed MSCP had a very fast photoresponse rate. The rise time was 74 μs and the fall time was 58 μs, around three orders of magnitude faster than those measured in MPFP (52 ms for rise time and 36 ms for fall time in this work or about hundred ms according to the literature^19^). After 40 days’ storage in a desiccator at room temperature without any modification, the MSCP device still kept a high photocurrent value of 2.65 μA that only fell by 6%. In addition, further enhancements of photodetector performance could be expected by improving the interface between the perovskites and electrodes. These results pave the way for exploiting high-performance perovskites photodetectors based on single crystal.

## Methods

### Single Crystal Growth

Seed crystals were prepared according to Poglitsch and Weber’s method^27^. 5g lead(II) acetate trihydrate (99.5%, Sinoreagent) was dissolved in 20 ml hydroiodic acid (57 wt.% aqueous solution, ACROS ORGANICS) through an ultrasound treatment. 1.33 ml methylamine (40% w/w aqueous solution, Aladdin) was dissolved in 4 ml hydroiodic acid solution. The two solutions were heated to 373 K respectively. They were kept at that temperature for 2 hours, and then they mixed. Small angular black crystals about 3 mm in size were obtained by cooling down the solution from 373 K gradually to 330 K for 5 days. These small crystals were drawn out of the solution above 320 K and served as seed crystals for the subsequent bulk single crystal growth. Harvesting at temperature below 310 K would be harmful to the completeness of the seed crystals since it would incur the formation of acicular (MA)_4_PbI_6_·2H_2_O crystals^27^,^37^. Bulk single crystals of MAPbI_3_ were grown via the bottom-seeded solution growth method. A carefully selected seed crystal was introduced into the bottom of the growth solution by fixing it at the end of a Pt wire immersed in the growth solution at 373 K. The temperature was kept 373 K for 10 min to dissolve the outer surface of the seed crystal, then it quickly dropped to 355 K and followed by a slow cooling rate until the desired size was obtained. Subsequently, it dropped to 330 K at a slow cooling rate of 5 K per hour. Ultimately, the resultant rhombic dodecahedral MAPbI_3_ bulk single crystal was taken out from the growth solution at a temperature higher than 320 K.

### Characterization of MAPbI_3_ Single Crystal

Single crystal XRD data were collected on a Xcalibur, Eos, Gemini device at 293(2) K using Mo Kα radiation (λ = 0.7107 Å). All calculations were performed with programs from the SHELXTL crystallographic software package^38^. Final least-squares refinement on F_o_^2^ with data having F_o_^2^ ≥ 2σ(F_o_^2^) including anisotropic displacement parameters for all nonhydrogen atoms. The structure was checked for missed symmetry elements on the model using PLATON^39^. The single crystal plates for optical characterization and device fabrication were obtained by cutting from the oriented bulk single crystal followed by polishing to 1 mm in thickness. The as-prepared single crystal plates were oriented by X-ray diffraction (XRD) spectroscopy (Brüker D8 Advance) operated with Cu Kα radiation at 40 kV and 40 mA. X-ray crystal diffraction instrument (DX-2/4A) was also used to confirm the orientation. The transmission spectrum was collected on a Lambda 950UV-Vis-NIR spectrophotometer (PerkinElmer).

### Device Fabrication

Planar-type MSCP devices were fabricated by depositing 140 nm interdigital gold-film electrodes via vacuum evaporation method on the (100) facet of MAPbI_3_ single crystal plate. The bridging-gap of electrodes width is about 20 μm, while the effective illuminated area of MAPbI_3_ single crystal was about 1.19 × 10^−7^ m^2^. While the bare MAPbI_3_ film was prepared according to Seok’s method^40^. CH_3_NH_3_I (MAI) was synthesized by mixing 27.86 ml CH_3_NH_2_ (40% in methanol, Aladdin) and 30 ml HI (57 wt% in water, ACROS ORGANICS) in a flask at 273 K with 4 h stirring. The precipitate was recovered by evaporation at 328 K for 1 h, then dissolved in ethanol, recrystallized from diethyl ether, and dried at 333 K in a vacuum oven for 24 h. The prepared MAI, PbI_2_ (99.5%, Sinoreagent) for 0.8 M MAPbI_3_ solution were stirred in a mixture of GBL and DMSO (7:3 v/v) at 333 K for 12 h. The mixture was coated onto the mp-TiO_2_/bl-TiO_2_/FTO substrate, which was prepared in advance yet through Seok’s method, by a consecutive two-step spin-coating process at 1,000 r.p.m for 10 s. During the second step, the substrate (around 20 mm × 20 mm) was treated with toluene drop-casting. Then it was dried on a hot plate at 373 K for 10 min. Finally, an effective area of about 13 mm × 6 mm was cutted for further thermal evaporation of erelectrode. Then the planar-type MPFP devices were fabricated by depositing the identical interdigitated Au-film electrodes on the surface of MAPbI_3_ thin-film.

### Device Characterization

The wavelength-dependent photoresponse measurements were performed with a 300 W Xe lamp equipped with a manual monochromator. The photoresponse characteristics of as-fabricated MSCP were investigated in the range from 275 nm to 790 nm with an interval of 25 nm. A set of optical power meter was used to measure the incident powers. Besides, all the other irradiations were achieved by using a 532 nm semiconductor laser with a spot diameter of about 3.00 mm. The electrical characteristics except for the transient performances and irradiance power-dependent photoresponse characteristics were collected by a Keithley 2400 SourceMeter. The high speed features and irradiance power-dependent photoresponse characteristics of the device were measured by an optical chopper and a precision Source/Measure Unit (Agilent B2911A). For irradiance power-dependent photoresponse measurements, a polarization modulator, which composed of a fixed polarizer and a rotatable analyzer, was used in order to obtain the variety of irradiance power densities. A gold thin film was utilized to achieve the weakest light below 10^−5^ mW cm^−2^.

## Additional Information

**How to cite this article**: Lian, Z. *et al.* High-Performance Planar-Type Photodetector on (100) Facet of MAPbI_3_ Single Crystal. *Sci. Rep.* **5**, 16563; doi: 10.1038/srep16563 (2015).

## Supplementary Material

Supplementary Information
