# Supplementary material for: High-Performance Planar-Type Photodetector on (100) Facet of MAPbI3 Single Crystal
Source: Sci Rep. 2015 Nov 13;5:16563. doi: 10.1038/srep16563 (PMC4643309; doi:10.1038/srep16563)
Supplement: Supplementary Information [file srep16563-s1.doc]

Supplementary information

**High-Performance Planar-Type Photodetector on (100) Facet of MAPbI_3_ Single Crystal**

Zhipeng Lian^1^, Qingfeng Yan^1^, *, Qianrui Lv^1^, Ying Wang^2^, Lili Liu^2^, Lijing Zhang^1^, Shilie Pan^2^, Qiang Li^1^, Liduo Wang^1^, and Jia-Lin Sun^3^, *

^1^ Department of Chemistry, Tsinghua University, Beijing 100084, China

^2^ Key Laboratory of Functional Materials and Devices for Special Environments, Xinjiang Technical Institute of Physics & Chemistry, Chinese Academy of Sciences, 40-1 South Beijing Road, Urumqi 830011, China

^3^ Collaborative Innovation Center of Quantum Matter, State Key Laboratory of Low-Dimensional Quantum Physics, Department of Physics, Tsinghua University, Beijing 100084, China

*******Corresponding author: yanqf@mail.tsinghua.edu.cn; jlsun@tsinghua.edu.cn

**S1. Performance comparison of photodetectors based on pure MAPbI3 materials:**

**Table S1. A comparison of several recently reported pure MAPbI_3_** photodetectors.

| Device structure | Responsivity and effiency | Conditions | Response time | Ref |
| --- | --- | --- | --- | --- |
| Vertical type:  ITO/MAPbI_3_/TPD-Si_2_/MoO_3_/Ag | R: 84, 203, and 242 A W^-1^, respectively. | at three wavelengths of 350, 530, and 740 nm, respectively;  at low bias −1V | rise time: ~10 μs;  fall time:  ~46 μs | 1 |
| Planar-type:  ITO/MAPbI_3_/ITO | R:3.49 A W^-1^ ;  EQE(cal.): 1.19×10^3^ % | 365 nm light；0.01 mW cm^-2^,  at a bias of 3 V. | ＜200 ms | 2 |
|  | R: 0.0367 A W^-1^;  EQE(cal.): 5.84% | 780 nm light;  0.01 mW cm^-2^,  at a bias of 3 V; | ＜100 ms |  |
| Planar-type:  SiO_2_/Si/ODTS/Au/Graphene/MAPbI_3_ | R:180 A W^-1^;  effective quantum efficiency: 5 ×10^4^ % | 400 to 800 nm;  at 1 µW illumination. | rise time: 87 ms;  fall time:  540 ms | 3 |
|  | R:~0.5 A W^-1^ | 400 to 800 nm;  at 1 mW illumination. |  |  |
| **Planar-type:**  **Au/ Polycrystalline film of MAPbI_3_/Au** | **R: 0.28 A W^-1^**  **EQE(cal.):64.64%** | **at 1 V with 532 nm 120 nW cm^-2^ irradiance** | **rise time: 52 ms;**  **fall time:**  **36 ms** | **This**  **work** |
|  | **R: 0.0197 A W^-1^**  **EQE(cal.):4.59%** | **at 1 V with 532 nm 1 mW cm^-2^ irradiance** |  |  |
| **Planar-type:**  **Au/~~MSC~~ Single Crystal of MAPbI_3_/Au** | **R: 953 A W^-1^**  **EQE(cal.):2.22 × 10^5^ %** | **at 1 V with 532 nm 2.12 nW cm^-2^ irradiance** | **rise time: 74** **μs;**  **fall time:**  **58 μs** | **This**  **work** |
|  | **R: 2.55 A W^-1^**  **EQE(cal.):5.95 × 10^2^ %** | **at 1 V with 532 nm 1 mW cm^-2^ irradiance** |  |  |

Note：EQE represents external quantum efficiency; MSC represents MAPbI_3_ single crystal.

**S2. Structure analysis of MAPbI_3_ crystal.**

For MAPbI_3_, there are one lead atom, one carbon atom, one nitrogen atom, and two iodine atoms in the asymmetric unit. Pb atom is coordinated by two types and six I atoms, forming distorted PbI_6_ octahedra. C atom is surrounded by four disordered N atoms. As shown in **Figure S1**a, MAPbI_3_ features a three-dimensional Pb-I framework which is built up by Pb-I layers (Figure S1b) connected by I(1) atoms linkers. The Pb-I layer is the result of extension of PbI_6_ octahedron on the a-b plane via sharing I(2) atoms (Figure S1b). Figure S1a can be simplified as Figure S1c, where the green and violet lines represent adjacent two layers, respectively. Down to c-axis, we can get that adjacent layers arrange crisscross with each other (Figure S1d). MA^+^ groups are put into the square space of Pb-I framework to form the whole MAPbI_3_ structure (**Figure S2**). **Table S2** provides the single crystal data and structure refinement information.


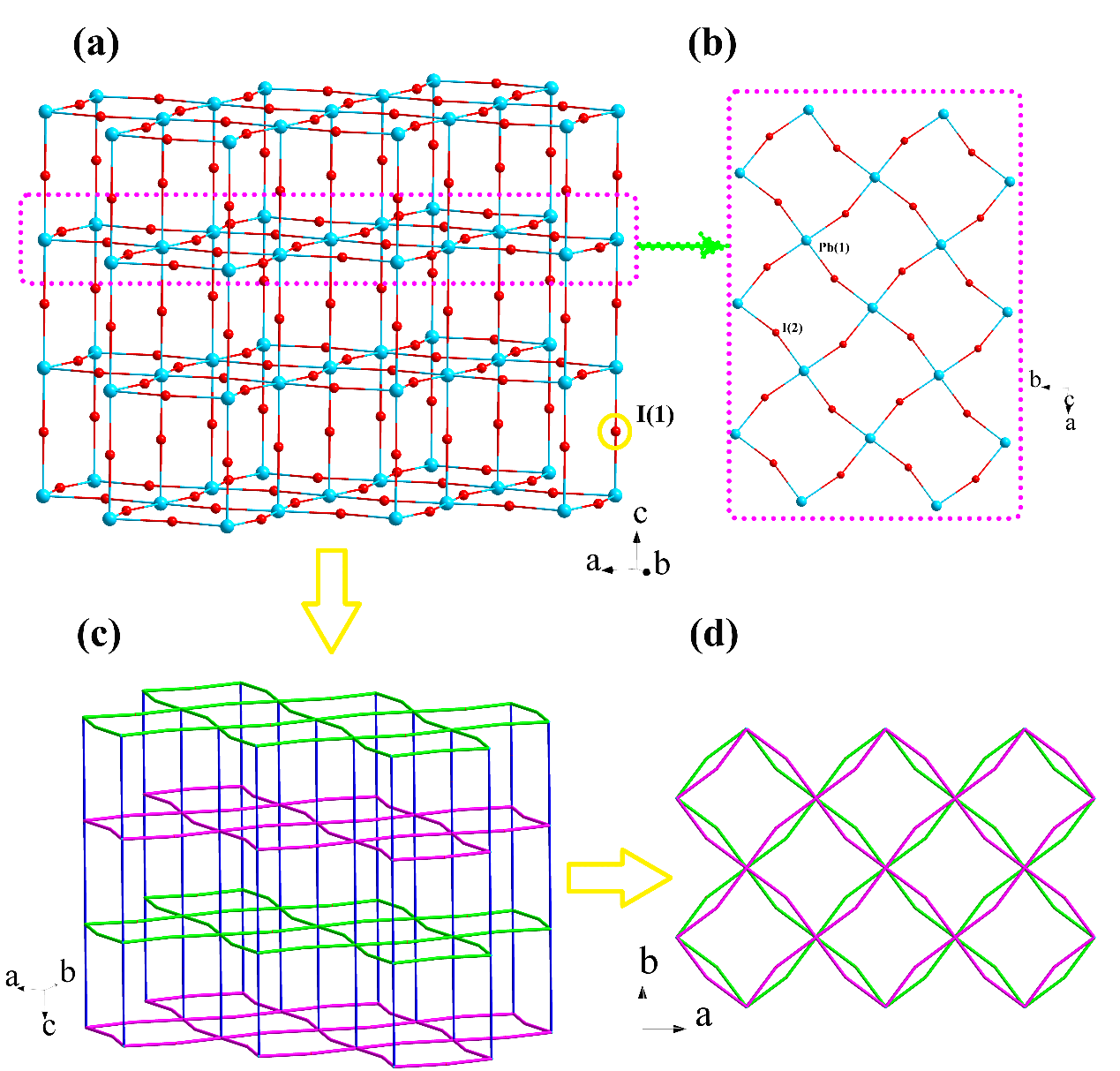


**Figure S1.** **Structure drawing of Pb-I skeletons.**


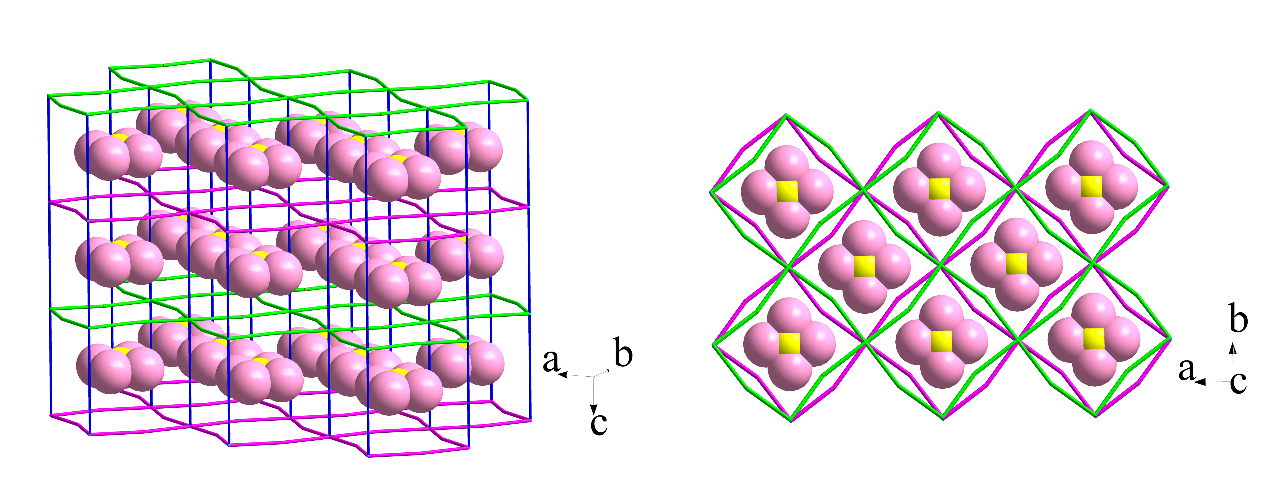


**Figure S2.** **The position of MA^+^ in Pb-I framework.**

**Table S2.** Crystal data and structural refinements.

| compound | MAPbI_3_ |
| --- | --- |
| formula weight (g mol^-1^) | 619.96 |
| temperature (K) | 293 (2) |
| crystal system  space group | Tetragonal |
|  | I4/mcm |
| *a* (Å) | 8.8429(5) |
| *b* (Å) | 8.8429(5) |
| *c* (Å) | 12.6255(10) |
| *α (deg)* | 90.00 |
| β (deg) | 90.00 |
| γ (deg) | 90.00 |
| volume (Å^3^) | 987.28(11) |
| Z | 4 |
| D_calcd_ (g cm^-3^) | 4.130 |
| absorption coeffient (mm^-1^) | 26.384 |
| Crystal size (mm^3^) | 0.15 × 0.15 × 0.12 |
| GOF on *F*^2^ | 1.160 |
| R_1_, *w*R_2_ [*F*_o_^2^>2σ( *F*_o_^2^)]*^a^* | 0.1473 |
| R_1_, *w*R_2_ (all data) *^a^* | 0.0572 |
| Min/max Δρ (e Å^-3^) | -3.012/2.913 |

*^a^* R_1_ = Σ*F*_o_ - *F*_c_/Σ*F*_o_ and *w*R_2_ = [Σw(*F*_o_^2^ – *F*_c_^2^)2/ Σw *F*_o_^4^]^1/2^ for *F*_o_^2^ > 2σ( *F*_o_^2^).

**S3. SHG Measurement.**

In order to further understand the structure of MAPbI_3_, the second harmonic generation (SHG) response was measured by using the Kurtz and Perry method with 2.05 μm Q-switch laser.^4,5^ The required MAPbI_3_ powder samples were obtained by grinding the MAPbI_3_ crystals thoroughly in an agate mortar. Condition of environment humidity was strictly controlled to avoid the deliquescence of the powder samples. However, no SHG signal was observed, implying that the space group of MAPbI_3_ was more reliable to be centrosymmetric I4/mcm, rather than the noncentrosymmetric one of I4cm.

**S4. Optical transmission characterization.** Optical properties were investigated by using transmission spectrum. Firstly, a single crystal plate of 1 mm in thickness was fabricated by cutting a (100)-oriented MAPbI_3_ single crystal followed by polishing. **Figure S3** shows the photograph of a thin plate of MAPbI_3_ single crystal with a thickness of 1 mm. In order to deduce band-gap of MAPbI_3_ single crystal, transmission spectrum was collected by using the as-prepared MAPbI_3_ (100)-cut plate (**Figure S4**). As shown in the inset of **Figure S4**, the optical band-gap of MAPbI_3_ single crystal was calculated through the extrapolation method to be 1.48 eV.


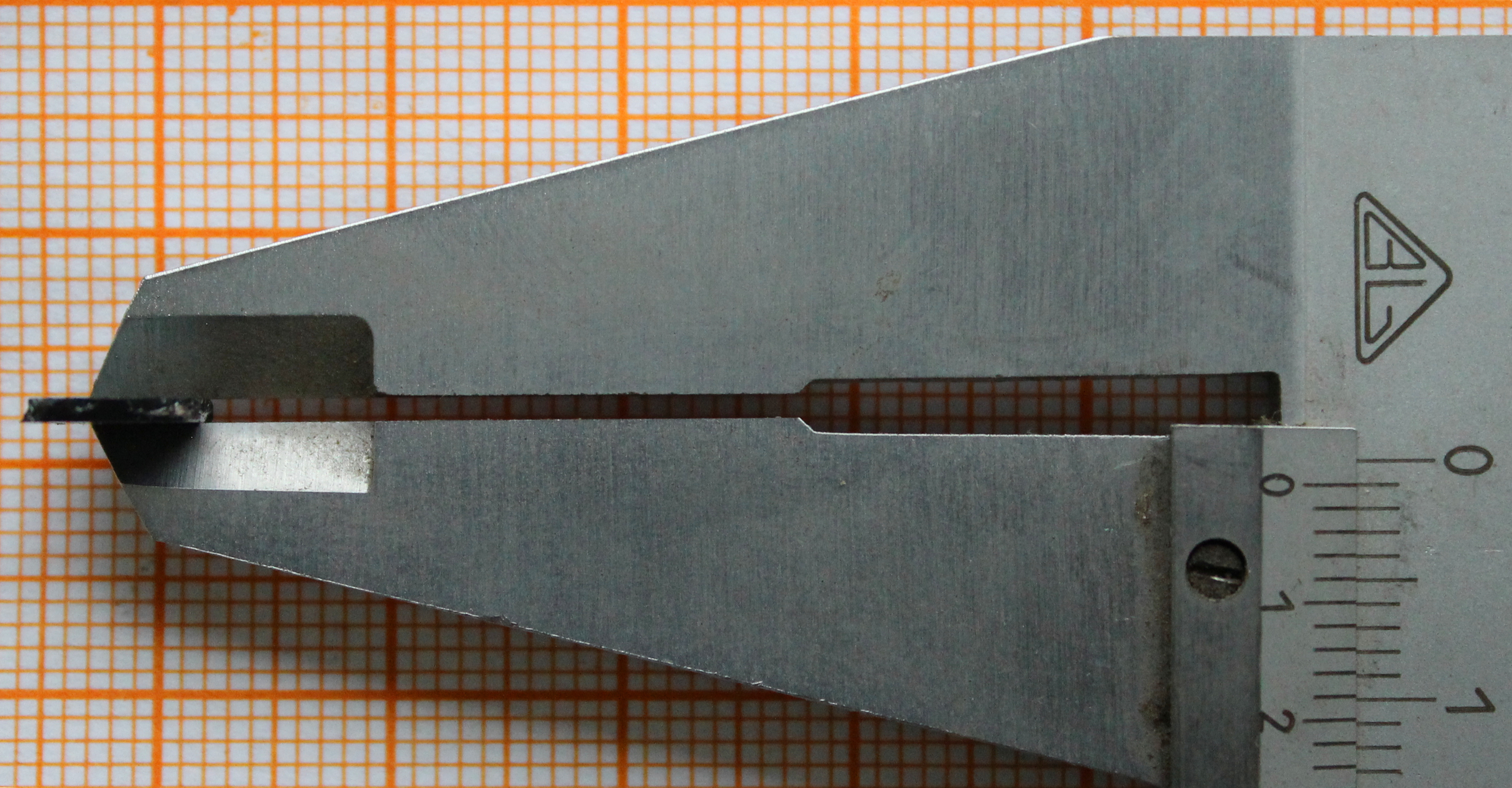


**Figure S3.** **Photograph of (100)-cut MAPbI_3_ single crystal plate with a thickness of 1 mm.**

**Figure S4.** **Transmission spectrum of as-prepared** **MAPbI_3_ (100)-cut single crystal plate.** The inset shows the corresponding absorption spectrum and band-gap calculation.

**S5. Raman measurement.**

Vibrational investigation of a (100)-facet 1 mm-thick plate of MAPbI_3_ single crystal was performed by Raman spectra, with a confocal microscope Raman spectrometer (HORIBA JY HR800) employing an Ar–ion laser operating at 514 nm. The output power was kept below about 300 μW on the sample to avoid any sample degradation. A 50× telephoto Olympus objective lens was employed to focus the laser on the samples with laser spot size of ca. 2 μm. The spectra were collected in the range of 50 to 400 cm^-1^ and the system was calibrated with respect to a silicon wafer at 520.7 cm^-1^. All the measurements were performed at room temperature. An excellent study on Raman spectrum for a MAPbI_3_ polycrystalline film deposited on mesoporous Al_2_O_3_ has been reported theoretically and experimentally,^6^ which provides useful guidelines for single crystal investigation. **Figure S8** shows the Raman vibrational spectra of the MAPbI_3_ single crystal in the low-frequency region at room temperature. The Raman spectra presents various bands between 50 and 300 cm^-1^, peaking at 74, 96, 110, 166, and 216 cm^-1^. The band at 74 cm^-1^ can be assigned to the libration of I−Pb−I bonds and the deformation of the inorganic cage.^7,8^ The 96 cm^-1^ band can be ascribed to both the Pb−I stretching and the libration modes of the cations, while the inorganic component plays a major role. The bands peaking at 110 and 166 cm^-1^ are probably associated to the libration of the organic cations.^9^ Further, the band at 216 cm^-1^ may correspond to the torsional mode of MA^+^ cations.^6^

**Figure S5.** **Raman spectra of MAPbI_3_ crystal.**

**S6. Electrode structure of the MSCP.**

An optical micrograph of the planar-type photodetector fabricated on the (100) facet of a MAPbI_3_ single crystal was shown in **Figure S6**. Two adjacent interdigital Au film electrodes was deposited on the (100)-facet of MAPbI_3_ single crystal. The bridging gap between the Au conductive electrodes was about 20 µm, while the effective illuminated area was about 1.19×10^-7^ m^2^.


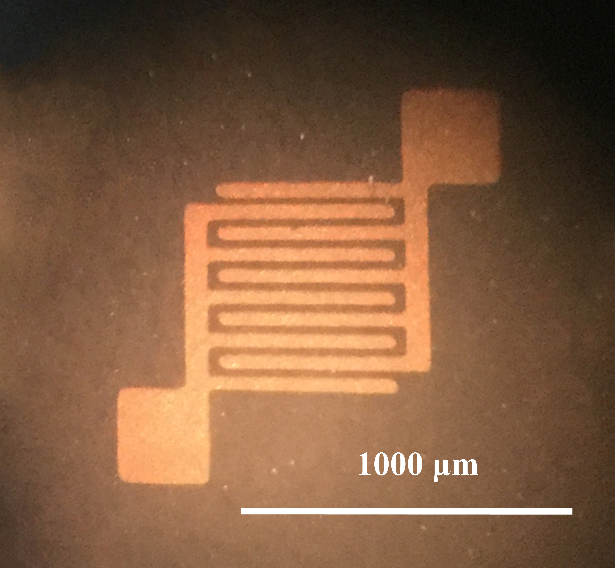


**Figure S6**. **Optical micrograph of the** **planar-type MSCP.**

**S7. Charging and discharging behaviors observed in MSCP.**

As seen in Figure S7, when an electric supply of 1 V was triggered, an instant photocurrent as high as 3.57 μA was obtained. Then the photocurrent decayed as a function of time until an equilibrium of 1.52 μA was reached. As soon as the power supply was cut off, the device outputted a reverse current as high as 1 μA. As time passed, the reverse current gradually diminished and the build-in internal electric field wore off. The decay and recovery time, both defined as the time taken from the initial current value, decrease 90% to the equilibrium, were calculated to be 17.6 s under 1 V bias, and 29.5 s after cutting-off bias, respectively. These results suggest the existence of the built-in electric fields in MSCP. However, the accurate reasons for such an effect remain to be explored in further investigation.

**Figure S7**. **Charging and discharging behaviors observed when applying a switching on-and-off of 1 V electric supply under 532 nm 0.79 mW/cm^2^ laser illumination.**

**S8. Device photographs of as-fabricated MSCP and MPFP.**

As shown in the insets in Figure S8, identical interdigitated Au-film electrodes was deposited on (100) facet of MAPbI_3_ single crystal and the surface of MAPbI_3_ thin-film, respectively. 25 μm gold wires were used to connect the Au-film electrodes with the test beds.

**
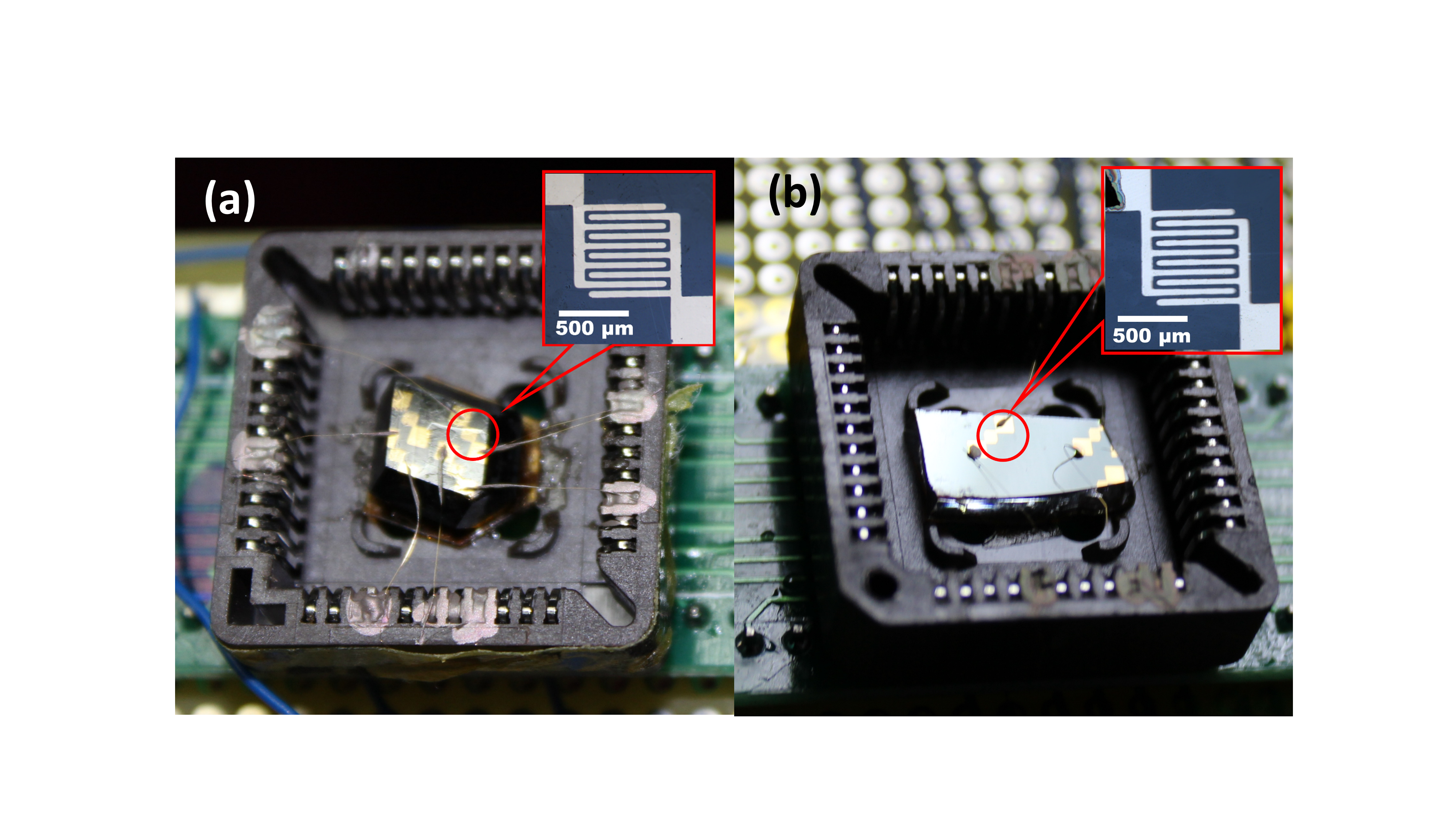
**

**Figure S8**. **The photographs of the MSCP (a) and MPFP (b) devices.** The insets show the optical micrograph for each device.

**S9. Irradiance-dependent photocurrent.**

At a bias of 1 V, the illumination power-dependent photocurrent was plotted in Figure S9 with the irradiance intensity varying from 2.12 nW cm^-2^ to 82.2 mW cm^-2^. The relationship between the photocurrent and the irradiance intensity can be fitted by the power law of *I*_ph_$=a\cdot P_{0}^{x}$, where *I*_ph_ represents photocurrent intensity, $a$ is constant, *P*_0_ represents irradiance power, and *x* is the exponential term. As a result, the irradiance-dependent photocurrent curve was fitted to be *I*_ph_ ~ *P*_0_^0.632^.

**Figure S9. Photocurrent versus irradiance power density (*P_0_*) plot at a bias of 3 V.**

**S10. LDR characterization.**

The linear dynamic range (LDR) is another figure of merit for photodetector, typically quoted in dB. Under 532 nm MSCP exhibited a linear response with light intensity varying from 2.12 nW cm^-2^ to 82.2 mW cm^-2^. LDR represents that the photocurrent has a linear response with the incident light intensity changes in a certain range, which is given by: ^3^

$$LDR=10\text{log}\frac{P_{\max}}{P_{\min}}$$

where$P_{\max}$ is is the highest incident light power and $P_{\min}$ is the lowest detectable incident light power of the range in which the photodetector response is linear with the incident power.. The LDR for our MSCP was calculated to be 76 dB. Moreover, the noise equivalent power (NEP), which represents the minimum impinging optical power that a detector can distinguish, could be estimated by LDR measurement method. Through gradually attenuating the irradiance power, as shown in Figure S9, the lowest detectable light intensity was measured to be 2.52 pW, indicating a very small NEP for MSCP.

**Figure S10. Linear dynamic range of the MSCP.**

**S11. Power spectrum of Xe lamp at different wavelengths.**

The wavelength-dependent photocurrent was measured by using a Xe lamp equipped with a manual monochromator. In order to evaluate the wavelength influence on the photocurrent and obtain the corresponding responsivity and EQE values at each selected wavelength ranging from 250 to 790 nm, multiple measurements of the incident optical powers were averaged and plotted in **Figure S11**.

**Figure S11.** **The irradiance power density (*P_0_*) measured at different wavelengths using an optical power meter.**

**References**

1. Dong, R. *et al.* High-Gain and Low-Driving-Voltage Photodetectors Based on Organolead Triiodide Perovskites. *Adv. Mater.* **27**, 1912-1918 (2015).
2. Hu, X. *et al.* High-Performance Flexible Broadband Photodetector Based on Organolead Halide Perovskite. *Adv. Funct. Mater.* **24**, 7373-7380 (2014).
3. Lee, Y. *et al.* High-Performance Perovskite-Graphene Hybrid Photodetector. *Adv. Mater.* **27**, 41-46 (2015).
4. Kurtz, S. & Perry, T. A powder technique for the evaluation of nonlinear optical materials. *J. Appl. Phys.* **39**, 3798-3813 (1968).
5. Kurtz, S., Perry, T. & Bergman Jr, J. Alpha-Iodic Acid: A Sollution-Grown Crystal For Nonlinear Optical Studies and Applications. *Appl. Phys. Lett.* **12**, 186-188 (1968).
6. Quarti, C. *et al.* The Raman spectrum of the CH_3_NH_3_PbI_3_ hybrid perovskite: interplay of theory and experiment. *J. Phys. Chem. Lett.* **5**, 279-284 (2013).
7. Dammak, T., Fourati, N., Boughzala, H., Mlayah, A. & Abid, Y. X-ray diffraction, vibrational and photoluminescence studies of the self-organized quantum well crystal H_3_N(CH_2_)_6_NH_3_PbBr_4_. *J. Lumin.* **127**, 404-408 (2007).
8. Elleuch, S., Abid, Y., Mlayah, A. & Boughzala, H. Vibrational and optical properties of a one-dimensional organic-inorganic crystal [C_6_H_14_N]PbI_3_. *J. Raman Spectrosc.* **39**, 786-792 (2008).
9. Maalej, A. *et al.* Phase transitions and crystal dynamics in the cubic perovskite CH_3_NH_3_PbCl_3_. *Solid State Commun.* **103**, 279-284 (1997).
10. Liu, J. *Photonic devices*. (Cambridge University Press, 2005).
